# Development of a NIR Iridium(III) Complex-Based Probe for the Selective Detection of Iron(II) Ions

**DOI:** 10.3390/bios14080369

**Published:** 2024-07-29

**Authors:** Wanyi Wang, Zixi Zhang, Jingqi Liu, Lingtan Kong, Wanhe Wang, Chung-Hang Leung, Jing Wang

**Affiliations:** 1 Xi’an Key Laboratory of Stem Cell and Regenerative Medicine, Institute of Medical Research, Northwestern Polytechnical University, 127 West Youyi Road, Xi’an 710072, China; wanyiw@mail.nwpu.edu.cn (W.W.); zzixi@mail.nwpu.edu.cn (Z.Z.); liujingqi715@mail.nwpu.edu.cn (J.L.); klt1030@mail.nwpu.edu.cn (L.K.); whwang0206@nwpu.edu.cn (W.W.); 2 Research & Development Institute, Northwestern Polytechnical University in Shenzhen, 45 South Gaoxin Road, Shenzhen 518057, China; 3 State Key Laboratory of Quality Research in Chinese Medicine, Institute of Chinese Medical Sciences, University of Macau, Taipa, Macau, China; 4 Department of Biomedical Sciences, Faculty of Health Sciences, University of Macau, Taipa, Macau, China; 5 Macao Centre for Research and Development in Chinese Medicine, University of Macau, Taipa, Macau, China; 6 MoE Frontiers Science Center for Precision Oncology, University of Macau, Taipa, Macau, China

**Keywords:** iridium(III) complex, iron(II) ions, NIR detection, anti-interference ability, high sensitivity

## Abstract

As a commonly used metal ion, iron(II) (Fe^2+^) ions pose a potential threat to ecosystems and human health. Therefore, it is particularly important to develop analytical techniques for the rapid and accurate detection of Fe^2+^ ions. However, the development of near-infrared (NIR) luminescence probes with good photostability for Fe^2+^ ions remain challenging. In this work, we report a novel iridium(III) complex-based luminescence probe for the sensitive and rapid detection of Fe^2+^ ions in a solution based on an Fe^2+^-mediated reduction reaction. This probe is capable of sensitively detecting Fe^2+^ ions with a limit of detection (LOD) of 0.26 μM. Furthermore, this probe shows high photostability, and its luminescence remains stable under 365 nm irradiation over a time period of 30 min. To our knowledge, this is first iridium(III) complex-based NIR probe for the detection of Fe^2+^ ions. We believe that this work provides a new method for the detection of Fe^2+^ ions and has great potential for future applications in water quality testing and human monitoring.

## 1. Introduction

Iron (Fe) is the second most abundant element in the Earth’s crust and is also an essential nutrient in the human body [1]. In fact, approximately half of the iron in the human body is in the form of Fe^2+^ ions and Fe^3+^ [2], which are involved in many important physiological processes in the human body [3], such as oxygen transfer [4], nucleotide synthesis [5], electron transfer [6], and enzymatic reactions [7]. However, the presence of excess redox-active free Fe catalyzes the generation of hydroxide anions and increases the production of reactive oxygen species (ROS) [8,9], and abnormal levels of Fe^2+^ ions are highly associated with diseases including cancer and inflammation [10]. Therefore, the amount of water-soluble fraction of Fe^2+^ ions in the body is an important indicator for estimating Fe-induced cellular damage. On the other hand, the release of large amounts of Fe-containing chemicals into the environment may lead to a range of environmental and ecological problems [11]. Therefore, the monitoring and detection of Fe^2+^ ion levels are crucial in protecting environmental and living systems.

Traditional analytical techniques have been widely developed for the detection of Fe^2+^ and Fe^3+^ ions, such as colorimetric [12,13], electrochemical [14], and fluorescent methods [15,16]. Due to the fact that Fe^2+^ ions are easily oxygenated to Fe^3+^ ions in aerobic water [17], and numerous biometallic ions can be competitive in binding to common ligands, it is still a challenge to develop probes for the highly selective detection of Fe^2+^ ions. Reaction-based fluorescence probes offer great potential in overcoming the limitations above, which combine Fe^2+^-mediated reactions and the advantages of fluorescent techniques, including high sensitivity, high specificity, simple operation, and a fast response time [18]. In particular, the Fe^2+^-mediated *N*-oxide reduction strategy, first reported by Nagasawa et al. [19], has been demonstrated as a reliable and selective method to construct Fe^2+^ ion fluorescence probes. However, most of these probes suffer from narrow Stokes shifts, poor photostability, or a short emission wavelength of below 650 nm.

Recently, NIR iridium(III) complexes have emerged as promising luminescence probes for chemosensing and bioimaging [20] due to their long emission lifetime, high photostability, large Stokes shift, and tunable emissions. Moreover, NIR iridium(III) complex-based probes are highly resistant to background interferences and provide more accurate detection results [21]. These merits offer the possibility of compensating the shortcomings of the traditional Fe^2+^ ion fluorescence probes. Although a considerable number of NIR iridium(III) complexes have been developed for the detection of metal ions, small molecules, and disease-related proteins [22,23,24,25,26,27], no NIR iridium(III) complex-based probes are available for the detection of Fe^2+^ ions.

**Scheme 1:**
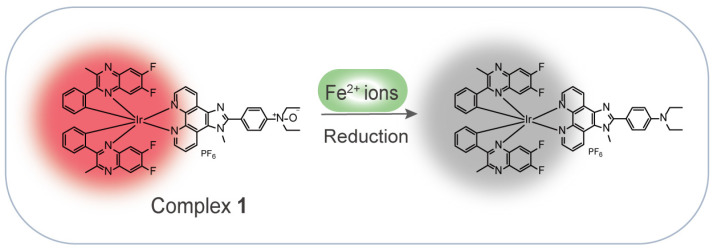
Schematic diagram of an iridium(III) complex-based NIR probe for the detection of Fe^2+^ ions.

In this work, inspired by the Fe^2+^-mediated *N*-oxide reduction reaction, we rationally designed and synthesized a NIR iridium(III) complex for the detection of Fe^2+^ ions in an aqueous solution (Scheme 1). Complex **1** showed a quenching luminescence upon the addition of Fe^2+^ ions through the Fe^2+^-mediated *N*-oxide moiety reduction of the N^N ligand. Moreover, the complex exhibited a good performance, with high selectivity and high sensitivity for the detection of Fe^2+^ ions, and a fast response of within 3 min.

## 2. Results and Discussion

### 2.1. Design and Synthesis of the Probe for Fe^2+^ Ions

**Scheme 2:**
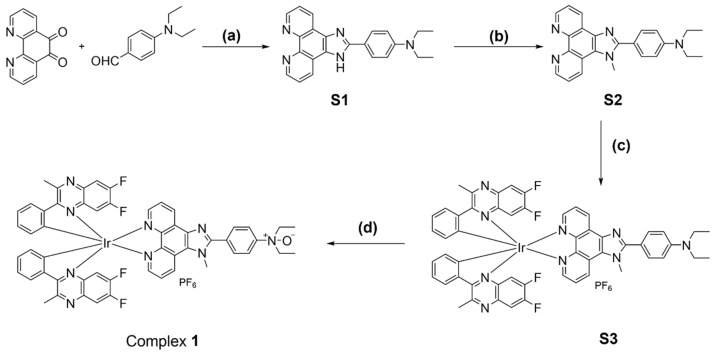
Synthesis of complex **1**. Reagents and conditions: (**a**) NH_4_OAc, HOAc, reflux, overnight, 87%. (**b**) *t*-BuOK, CH_3_I, THF, rt, overnight, N_2_, 56%. (**c**) Ir_2_(dfpq)_4_Cl_2_, DCM/MeOH, rt, overnight, then solid NH_4_PF_6_ added for another 0.5 h, rt, 58%. (**d**) *m*-CPBA, DCM, 0 °C to rt, 75%.

Fe^2+^ is a common divalent cation that can reduce oxides to their original elements or compounds [28]. This inspired us to design an oxide-containing NIR iridium(III) complex, in which the N^N ligand containing the *N*-oxide group was responsible for the specific recognition of Fe^2+^ ions, while 6,7-difluoro-2-methyl-3-phenylquinoxaline was chosen to act as C^N ligand to enable NIR emission (Scheme 1). It was expected that Fe^2+^ ions would trigger deoxygenation to generate complex **S3** and induce a luminescence quenching effect on complex **1**. Moreover, the weak complexation of Fe^2+^ ions with the O atom of the *N*-oxide might also promote the break of the *N*–*O* bond [29]. The complex was directly synthesized in four steps (Scheme 2): 1,10-phenanthroline-5,6-dione and *N*,*N*-diethylaminobenzaldehyde were cyclized under the catalyst of ammonium acetate (NH_4_OAc) to generate ligand **S1**, which was then substituted with lodomethane (CH_3_I) in the presence of potassium tert-butoxide (*t*-BuOK) to produce ligand **S2**. Ligand **S2** coordinated with the chloro-bridged iridium(III) dimer Ir_2_(dfpq)_4_Cl_2_ for the generation of complex **S3**, which was further oxidized by 3-chloroperoxybenzoic acid (*m*-CPBA) to afford the desired complex **1**. The purity of complex **1** was checked by high-performance liquid chromatography (HPLC), which showed a purity of over 95% (Figure S9). The intermediates and final complex were fully characterized by ^1^H NMR, ^13^C NMR, and electrospray ionization mass spectrometry (ESI-MS) (Figures S1–S8).

### 2.2. Photophysical Properties of the Probe

**Figure 1:**
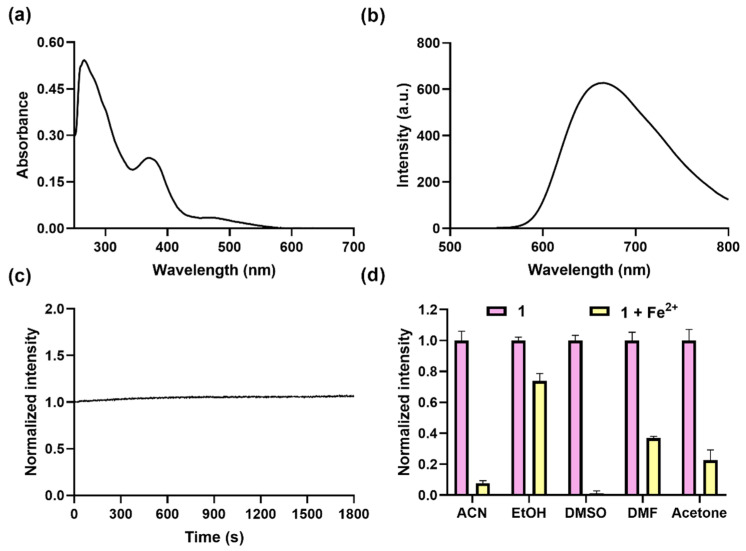
Optical profiles of complex **1** and its response to Fe^2+^ ions. Absorption (**a**) and luminescence spectra (**b**) of complex **1** (10 µM) in DMSO. (**c**) Time course of luminescence of complex **1** (10 µM) in DMSO over 1800 s under continuous irradiation at 365 nm. (**d**) Luminescence at 675 nm of complex **1** (10 μM) for Fe^2+^ (50 μM) in different solvents, including ACN, EtOH, DMSO, DMF, and acetone; λ_ex_ was set at 365 nm.

The absorption spectrum of complex **1** was first recorded in dimethyl sulfoxide (DMSO), which showed the assumed ligand-based π−π* transition peak at around 280 nm and a metal-to-ligand charge transfer (MLCT) transition peak at around 365 nm and 480 nm in DMSO (Figure 1a). The excitation and emission spectra showed that complex **1** had the maximum excitation wavelength of 365 nm and the maximum wavelength of 675 nm (Figure 1b and Figure S10). This indicates that complex **1** is a potential NIR probe and has a large Stokes shift of around 195 nm between the ^3^MLCT absorption and emission, which is much larger than organic dyes [30], making it less interfered with by the excitation light source. In order to assess the photostability, complex **1** was continuously irradiated over a time period of 1800 s at 365 nm (Figure 1c), showing no obvious decrease in luminescence intensity, which confirms its high photostability, ensuring reliable reproducibility and stability in the experimental results. 

Following the encouraging outcomes delineated above, we assessed the capacity of complex **1** in detecting Fe^2+^ ions across various solvent media. Complex **1** (10 μM) was added to various solutions, including ACN, ethanol (EtOH), *N*,*N*-dimethylformamide (DMF), DMSO, and acetone containing Fe^2+^ ions (50 μM), respectively; luminescence spectra showed that the best quenching effect was obtained in DMSO (Figure 1d), mainly due to cyclometalated iridium(III) complexes being lipophilic and having better solubility in DMSO and being slight soluble in alcohols [31,32]. In summary, complex **1** has the merits of a large Stokes shift, good photostability, NIR emission, and the best response to Fe^2+^ ions in DMSO, which is better than that for Fe^2+^ ion probes with an emission below 600 nm (Table S1).

### 2.3. Optimization of Detection Conditions

**Figure 2:**
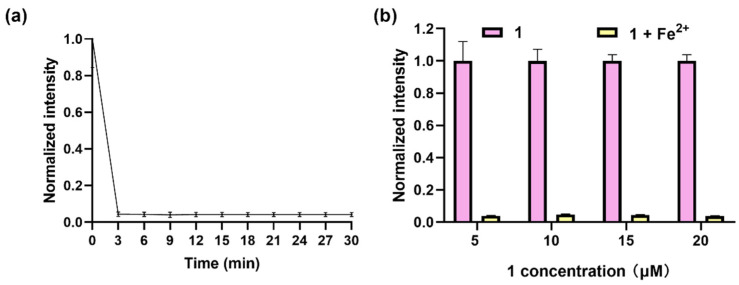
Optimization of detection parameters. (**a**) Luminescence of complex **1** (20 μM) for Fe^2+^ (50 μM) in DMSO within 30 min. (**b**) Luminescence of complex **1** (5–20 μM) with addition of Fe^2+^ (50 μM) in DMSO within 3 min. λ_ex_ was set at 365 nm, and λ_em_ was set at 675 nm.

In pursuit of optimal detection performance, a systematic optimization of both the incubation time and the concentration of the complex was conducted. Firstly, the luminescence response of complex **1** (20 μM) to Fe^2+^ (50 μM) over a time period of 30 min was investigated; the results showed that the luminescence dramatically dropped, reaching a plateau at 3 min (Figure 2a). Notably, this rapid response time contrasts with the behavior exhibited by several previously reported fluorescent probes (Table S1), which typically necessitate incubation periods of around 30 min or longer to achieve a plateau. Further, the luminescence response of complex **1** at different concentrations (5–20 μM) to Fe^2+^ (50 μM) within 3 min showed that the highest quenching effect was obtained at 20 μM (Figure 2b). Therefore, 3 min of incubation time and 20 μM of complex **1** were chosen for further experiments.

### 2.4. Sensitivity and Selectivity of Complex 1

**Figure 3:**
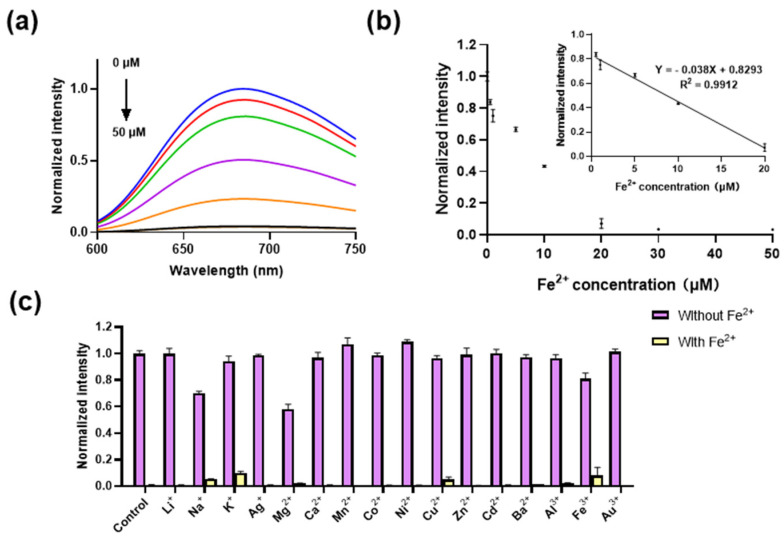
The sensitivity and selectivity of complex **1**. (**a**) Luminescence spectra of complex **1** (20 μM) with various concentrations of Fe^2+^ ions (0–50 μM) in DMSO. From blue to black lines represent different concentration of Fe^2+^ ions (0.5, 1,10, 20, 30, 50 μM). (**b**) Luminescence of complex **1** (20 μM) with various concentrations of Fe^2+^ ions (0.5–50 μM) in DMSO. Inset: The linear relationship between luminescence of complex **1** (20 μM) and Fe^2+^ ion concentrations (0.5–20 μM). (**c**) Luminescence of complex **1** (10 μM) for Fe^2+^ (50 μM) in the presence of various metal ions (500 μM) in DMSO. λ_ex_ was set at 365 nm, and λ_em_ was set at 675 nm.

After optimizing the reaction conditions, we investigated the luminescence response of complex **1** to varying concentrations of Fe^2+^ ions (0–50 μM). It is found that the luminescence of complex **1** decreased gradually with increasing concentrations of Fe^2+^ ions (Figure 3a, with a good linear relationship obtained between 0.5 and 20 μM (Figure 3b, Inset), obtaining a limit of detection (LOD) of 0.26 μM with a regression equation of Y = −0.038X + 0.8293 according to LOD = 3σ/k, and a sensitivity (*S_i_*) of −0.038. This is comparable to most of the reported fluorescence methods and better than the reported colorimetric methods (Table S1).

In order to assess the selectivity of complex **1** towards potentially interfering metal ions, the luminescence response of complex **1** (20 μM) to a range of metal ions in DMSO was investigated. The results demonstrated that the luminescent behavior of complex **1** remained consistent upon the introduction of other metal ions (at 10 equiv. concentrations, encompassing Li^+^, Na^+^, K^+^, Ag^+^, Mg^2+^, Ca^2+^, Mn^2+^, Co^2+^, Ni^2+^, Cu^2+^, Zn^2+^, Cd^2+^, Ba^2+^, Al^3+^, Fe^3+^, and Au^3+^ ions. Conversely, the subsequent addition of Fe^2+^ ions (50 μM) led to a substantial quenching of the luminescent signal emitted by the probe (Figure 3c). This indicates that the co-existence of other metal ions exerts a negligible influence over the luminescence of the complex toward Fe^2+^ ions, confirming its excellent selectivity. This attribute confers a distinct advantage over coordination-based Fe^2+^ ion probes (Table S1), as it minimizes the susceptibility to interference from various other metal ions.

### 2.5. Mechanism Study

**Figure 4:**
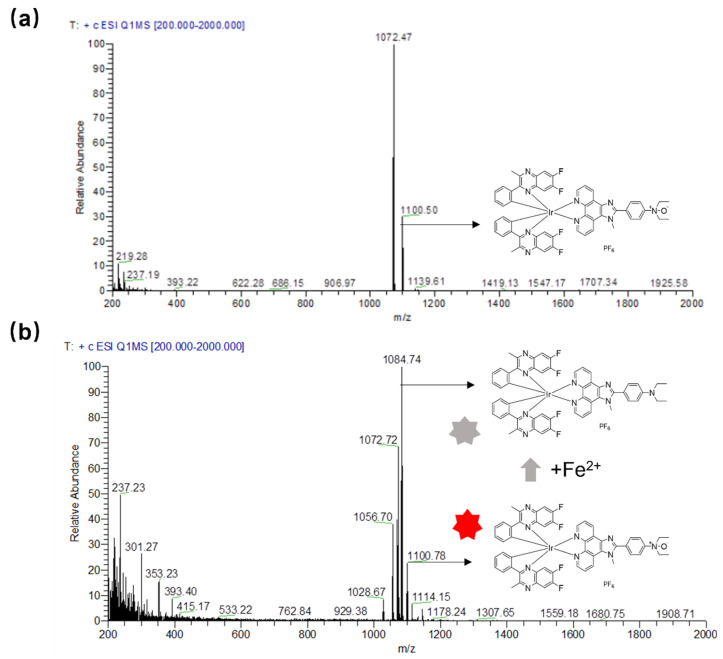
ESI-MS spectra of complex **1** (**a**) and complex **1** with the addition of Fe^2+^ ions (**b**). The colour change of the red to grey stars indicted the luminescence of complex **1** is quenched.

To validate the response mechanism of complex **1** for the detection of Fe^2+^ ions, ESI-MS was used to confirm the reduction of the *N*-oxide group by Fe^2+^ ions to afford control complex **S3**. The ESI-MS spectrum shows that the presence of Fe^2+^ ions induced a new *m*/*z* peak at 1084.30, corresponding to that of complex **S3**, while the *m*/*z* peak of complex **1** is 1100.74 (Figure 4). In addition, the reaction mechanism was further characterized by HPLC, and the result shows that the presence of Fe^2+^ ions induced the emergence of a peak with a residual time of around 18.60 min, compared with the residual time of about 14.00 min for complex **1** (Figure S11), which is consistent with complex **1** being more polar than complex **S3**. These results demonstrate that Fe^2+^ ions can reduce complex **1** to **S3**, and the reduction of the *N*-oxide group to an amino group presumably induces a photoinduced electron transfer (PeT) between the amino group and the iridium(III) complex, which transfers electrons from the donor to the excited state of the iridium(III) complex [33], leading to a strong luminescence quenching effect in complex **1**.

### 2.6. The Recovery by Complex 1 of Fe^2+^ Ions in Water Samples

The contamination associated with Fe^2+^ ions poses a significant threat to human health, making the practical application of complex **1** in environmental samples highly important. In this study, the feasibility of complex **1** in detecting Fe^2+^ ions in environment samples was investigated using water from 2% Xi’an Qixiang Lake as a model matrix. Complex **1** (20 μM) was mixed with Fe^2+^ ions at varying concentrations (5, 10, and 20 μM) in 2% lake-water-spiked DMSO, the recovery rates were found to range from 89.71% to 111.07%, with RSD values of 2.85% to 6.34% (Table S2). This result demonstrates the analytical potential of complex **1** in quantifying Fe^2+^ ions in environmental matrices.

## 3. Conclusions

In conclusion, we have successfully designed and synthesized a novel NIR luminescence probe for Fe^2+^ ion detection in aqueous solution. The complex can respond rapidly to Fe^2+^ ions within 3 min, exhibiting a high sensitivity, with an LOD of 0.26 μM. Moreover, the complex shows high selectivity for Fe^2+^ ions over other common metal ions, even in the presence of other interferents, and demonstrates a high photostability, with the luminescence remaining similar even under UV irradiation for 30 min. A further mechanism study demonstrates that the complex detects Fe^2+^ ions through Fe^2+^ ion-mediated *N*-oxide reduction, triggering a PeT process, along with luminescence quenching. In summary, this work provides a new analytical tool for the sensitive and selective detection of Fe^2+^ ions in aqueous solution, opening up a new avenue for the robust detection of Fe^2+^ ions. In future, we anticipate that leveraging advanced nanotechnological methodologies will facilitate the further development of this probe into a versatile array of tools [34]; these tools may encompass live imaging applications for the detection of Fe^2+^ ions within cancer systems, the targeted analysis of Fe^2+^ ions in cancerous tissues, and precision analyses dedicated to Fe^2+^ ion quantification in both untreated environmental specimens and blood samples.

## Data Availability

The original data involved in the study are included in the article, and further inquiries can be directly contacted by the corresponding author.

## Supplementary Materials

The following supporting information can be downloaded at https://www.mdpi.com/article/10.3390/bios14080369/s1, Figures S1–S8, NMR spectra of intermediates and complexes. Figure S9: HPLC analysis of complex **1** (Upper) and complex **1** in the presence of Fe^2+^ ions (Lower). The absorbance was detected at 254 nm. Figure S10: Excitation spectrum of complex **1** (10 μM) in DMSO. Table S1: Comparison of fluorescence probe/chemosensors for Fe^2+^ detection. Table S2: Recoveries of complex **1** (20 μM) for the detection of Fe^2+^ ions in DMSO containing 2% Xi’an Qixiang Lake water. References [35,36,37,38,39] are cited in the supplementary materials.
