# Supplementary material for: Development of a NIR Iridium(III) Complex-Based Probe for the Selective Detection of Iron(II) Ions
Source: Biosensors (Basel). 2024 Jul 29;14(8):369. doi: 10.3390/bios14080369 (PMC11352309; doi:10.3390/bios14080369)
Supplement: Supplementary file 1 [file biosensors-14-00369-s001.zip › biosensors-3090939-supplementary.pdf]

Supporting Information

Development of an NIR Iridium(III) Complex-Based Probe for the Selective Detection of Iron(II) Ions

Wanyi Wang [1,2,†], Zixi Zhang [1,2,†], Jingqi Liu [1,2,†], Lingtan Kong [1,2], Wanhe Wang [1,2], Chung-Hang Leung [3,4,5,6,*] and Jing Wang [1,2,*]

1 Xi'an Key Laboratory of Stem Cell and Regenerative Medicine, Institute of Medical Research,
Northwestern Polytechnical University, 127 West Youyi Road, Xi'an 710072, China;
wanyiw@mail.nwpu.edu.cn (W.W.); zzixi@mail.nwpu.edu.cn (Z.Z.);
liujingqi715@mail.nwpu.edu.cn (J.L.); klt1030@mail.nwpu.edu.cn (L.K.);
whwang0206@nwpu.edu.cn (W.W.)

2 Research & Development Institute, Northwestern Polytechnical University in Shenzhen,
45 South Gaoxin Road, Shenzhen 518057, China

3 State Key Laboratory of Quality Research in Chinese Medicine, Institute of Chinese Medical Sciences,
University of Macau, Taipa, Macau, China

4 Department of Biomedical Sciences, Faculty of Health Sciences, University of Macau, Taipa, Macau, China

5 Macao Centre for Research and Development in Chinese Medicine, University of Macau, Taipa,
Macau, China

6 MoE Frontiers Science Center for Precision Oncology, University of Macau, Taipa, Macau, China

* Correspondence: duncanleung@um.edu.mo (C.-H.L.); jwang0321@nwpu.edu.cn (J.W.)

† These authors contributed equally to this work.

Part A: Method and materials

Chemicals and reagents

1,10-Phenanthroline-5,6-dione purchased from Alpha Chemical Company, (Zhengzhou, China), 4-diethylaminobenzaldehyde was purchased from Bide Pharmatech Ltd. (Shanghai, China), Other reagents, unless specific stated, are purchased from J&K Scientific Company (Beijing, China).

Photophysical measurement

Luminescence emission spectra were recorded on a Hitachi F-4700 luminescence spectrophotometer at ambient temperature with an excitation wavelength at 375 nm. The excitation slit and emission slit were both set at 5.0 nm, PMT voltage was set at 500 V, with a scan speed of 1200 nm/min. UV−Vis absorption spectra were recorded on a Hitachi U-3900 UV–vis Spectrophotometer.

Part B: Synthesis experiment

Synthesis experiment

Mass spectrometry was performed at the Mass Spectroscopy Unit at the Institute of Medical Research, Northwestern Polytechnical University, Xi'an (China). Deuterated solvents for NMR purposes were obtained from Armar and used as received. ^{1}H and ^{13}C NMR were recorded on a Bruker Avance 500 spectrometer operating at 500 MHz (^{1}H) and 126 MHz (^{13}C) or a Bruker Avance 400 spectrometer operating at 400 MHz (^{1}H) and 101 MHz (^{13}C). ^{1}H and ^{13}C chemical shifts were referenced internally to solvent shift ($CDCl_3$-*d*: ^{1}H, 7.26, ^{13}C, 77.16; DMSO-d_6: ^{1}H, 2.50, ^{13}C, 39.52.). Chemical shifts are quoted in ppm, the downfield direction being defined as positive. Uncertainties in chemical shifts are typically ±0.01 ppm for ^{1}H and ±0.05 for ^{13}C. Coupling constants are typically ±0.1 Hz for ^{1}H−^{1}H and ±0.5 Hz for ^{1}H−^{13}C couplings. The following abbreviations are used for convenience in reporting the multiplicity of NMR resonances: s, singlet; d, doublet; t, triplet; q, quartet; m, multiplet; br, broad. All NMR data were acquired and processed using standard Bruker software (Topspin).

Synthesis of compound S1

A mixture of 1,10-phenanthroline-5,6-dione (1 eq), 4-diethylaminobenzaldehyde (1.1 eq), and ammonium acetate (NH_4OAc) (20 eq) in acetic acid (HOAc, 10 mL) was refluxed overnight. The reaction mixture was cooled to room temperature and dilute with water, then adjust the pH value of solution with sodium hydroxide (NaOH). The precipitate was filtered, and the resulting aqueous solution was extracted by dichloromethane (DCM) three times, then combined organic layer was dried over anhydrous sodium sulfate (Na_2SO_4), and the volatiles removed in vacuo. The residue was purified by silica column chromatography to obtain the product. Yield: 87%. ^{1}H NMR (500 MHz, Chloroform-*d*) δ 8.85 (dd, J = 8.2, 1.7 Hz, 2H), 8.79 (dd, J = 4.3, 1.7 Hz, 2H), 8.09 – 8.05 (m, 2H), 7.32 (dd, J = 8.2, 4.4 Hz, 2H), 6.42 – 6.38 (m, 2H), 5.29 (s, 0H), 3.15 (q, J = 7.0 Hz, 4H), 2.32 (s, 1H), 0.98 (t, J = 7.1 Hz, 6H). ^{13}C NMR (126 MHz, Chloroform-d) δ 147.39 , 143.53 , 130.62 , 128.37 , 123.10 , 111.21 , 44.32 , 12.62. ESI-MS: Calcd. for $C_{23}H_{21}N_5$ [M + H]: 367.18. Found: 368.19.

Synthesis of compound S2

A solution of compound **S1** (1 eq), lodomethane (CH_3I) (1.5 eq) and potassium *tert*-

butoxide (t-BuOK) (1.5 eq) in tetrahydrofuran (THF) was stirred at room temperature overnight under a nitrogen atmosphere. The solvent was removed under reduced pressure, and the crude product was purified by silica column chromatography to obtain the product. Yield: 56%. ^{1}H NMR (500 MHz, Chloroform-*d*) δ 9.17 – 9.03 (m, 3H), 8.71 (dd, J = 8.4, 1.7 Hz, 1H), 7.74 – 7.58 (m, 4H), 6.84 – 6.75 (m, 2H), 4.27 (s, 3H), 3.44 (q, J = 7.1 Hz, 4H), 1.22 (t, J = 7.0 Hz, 6H). ^{13}C NMR (126 MHz, Chloroform-d) δ 155.17, 148.83, 148.72, 147.50, 144.58, 144.08, 131.10, 130.61, 127.91, 126.04, 124.13, 123.46, 122.42, 120.45, 111.51, 44.60, 36.06, 12.68. ESI-MS: Calcd. for $C_{24}H_{23}N_5$[M + H]: 381.20. Found: 382.13.

Synthesis of compound S3

A solution of compound **S2** (2 eq) and the dichloro-bridged $Ir_2(dfpq)_4Cl_2$ (1 eq) in dichloromethane (CH_2Cl_2)(3 mL) and methanol (3 mL) was stirred at room temperature overnight. After the reaction completed, an excess of solid NH_4PF_6 was added and stirred for another 0.5 h. The solvent was removed under reduced pressure and purified by silica gel column chromatography to afford compound **S3**. Yield: 58%. ^{1}H NMR (400 MHz, DMSO-d_6) δ 9.23 (d, *J* = 8.2 Hz, 1H), 9.12 (dd, *J* = 8.2, 1.1 Hz, 1H), 8.61 – 8.55 (m, 4H), 8.15 (ddd, *J* = 7.8, 5.2, 2.4 Hz, 2H), 8.01 – 7.94 (m, 2H), 7.57 (d, *J* = 8.9 Hz, 2H), 7.35 (t, *J* = 7.7 Hz, 2H), 7.08 (s, 1H), 7.04 (dd, *J* = 11.9, 3.9 Hz, 1H), 6.97 (d, *J* = 8.1 Hz, 2H), 6.80 (dd, *J* = 11.8, 8.5 Hz, 4H), 4.22 (s, 3H), 3.61 – 3.56 (m, 2H), 3.44 – 3.39 (m, 6H), 1.13 (t, *J* = 7.0 Hz, 6H). ^{13}C NMR (101 MHz, DMSO) δ 165.10, 165.02, 159.77, 156.97, 154.32, 153.85, 153.36, 145.48, 144.78, 144.74, 144.69, 143.60, 143.58, 137.65, 137.34, 137.23, 136.20, 135.72, 133.60, 133.25, 132.13, 131.52, 131.41, 127.90, 127.27, 127.10, 125.78, 123.76, 122.46, 115.98, 111.39, 36.33, 27.45, 25.59, 12.84. ESI-MS: Calcd. For $C_{54}H_{41}F_4IrN_9$ $[M - PF_6]^+$: 1084.31. Found:1084.30

Synthesis of complex 1

3-Chloroperoxybenzoic acid (*m*-CPBA) was added slowly at 0 °C to a solution of complex **S3** in DCM. Then the mixture was stirred under rt for 4 h. The solvent was removed under reduced pressure and purified by silica gel column chromatography to afford final complex. Yield: 75%. ^{1}H NMR (400 MHz, DMSO-d_6) δ 9.31 (dd, J = 8.7, 1.3 Hz, 1H), 9.14 (dd, J = 8.3, 1.4 Hz, 1H), 8.61 (ddt, J = 21.6, 8.4, 1.6 Hz, 4H), 8.20 (dt, J = 8.3, 5.3 Hz, 2H), 8.13 – 7.93 (m, 6H), 7.36 (ddt, J = 8.6, 7.1, 1.5 Hz, 2H), 7.09 (dd, J = 12.4, 8.0 Hz, 1H), 7.05 – 6.94 (m, 3H), 6.79 (dt, J = 7.8, 1.5 Hz, 2H), 4.32 (ddt, J = 10.0, 7.0, 3.0 Hz, 2H), 4.26 (s, 3H), 4.20 (p, J = 6.8 Hz, 3H), 3.38 (d, J = 4.1 Hz, 6H), 1.12 (t, J = 7.0 Hz, 6H). ^{13}C NMR (101 MHz, DMSO) δ 165.12, 155.71, 154.36, 153.82, 153.65, 153.15, 152.65, 152.52, 152.11, 150.21, 149.76, 149.66, 148.28, 147.07, 146.87, 146.16, 146.00, 144.74, 144.23, 143.92, 137.79, 137.78, 137.58, 137.44, 137.36, 136.05, 133.72, 133.49, 132.41, 132.17, 132.13, 131.85, 131.56, 131.47, 131.42, 131.11, 129.26, 128.37, 128.31, 127.68, 127.40, 125.94, 123.81, 122.39, 122.31, 116.04, 110.51, 99.76, 66.35, 40.59, 36.25, 27.44, 8.35.ESI-MS: Calcd. for $C_{54}H_{41}F_4IrN_9O$ $[M - PF_6]^+$:1100.30. Found:1100.74

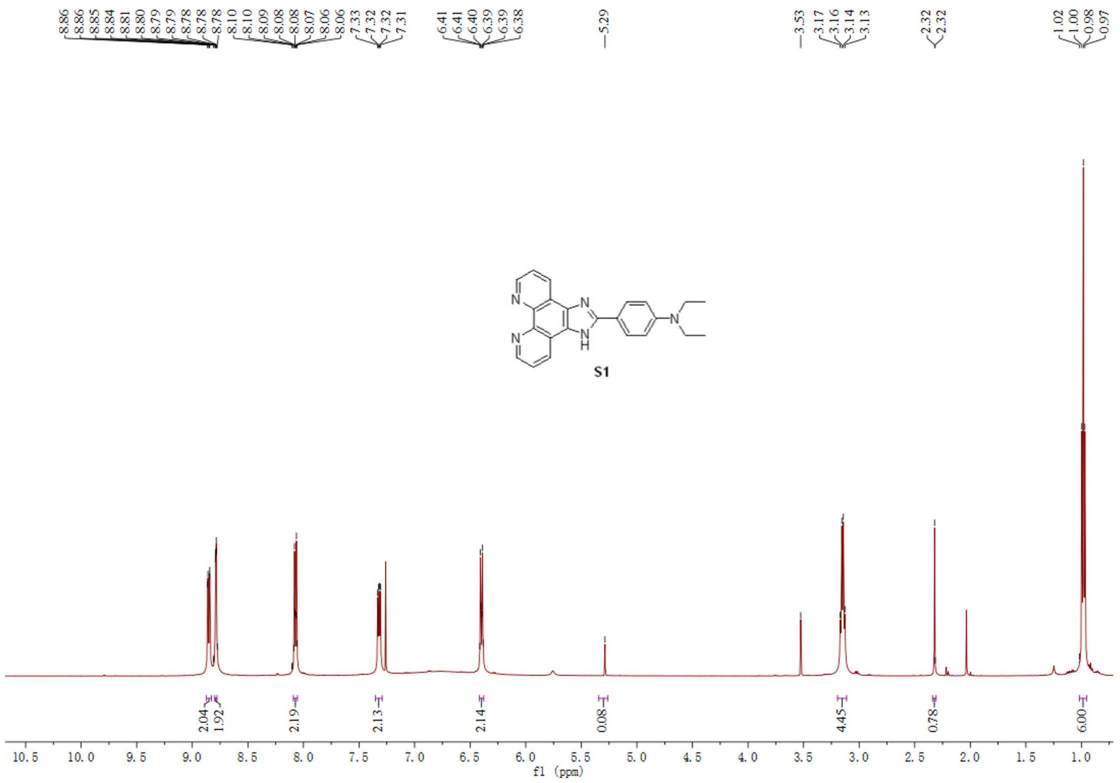

Figure S1. ^{1}H NMR spectrum of compound **S1**.

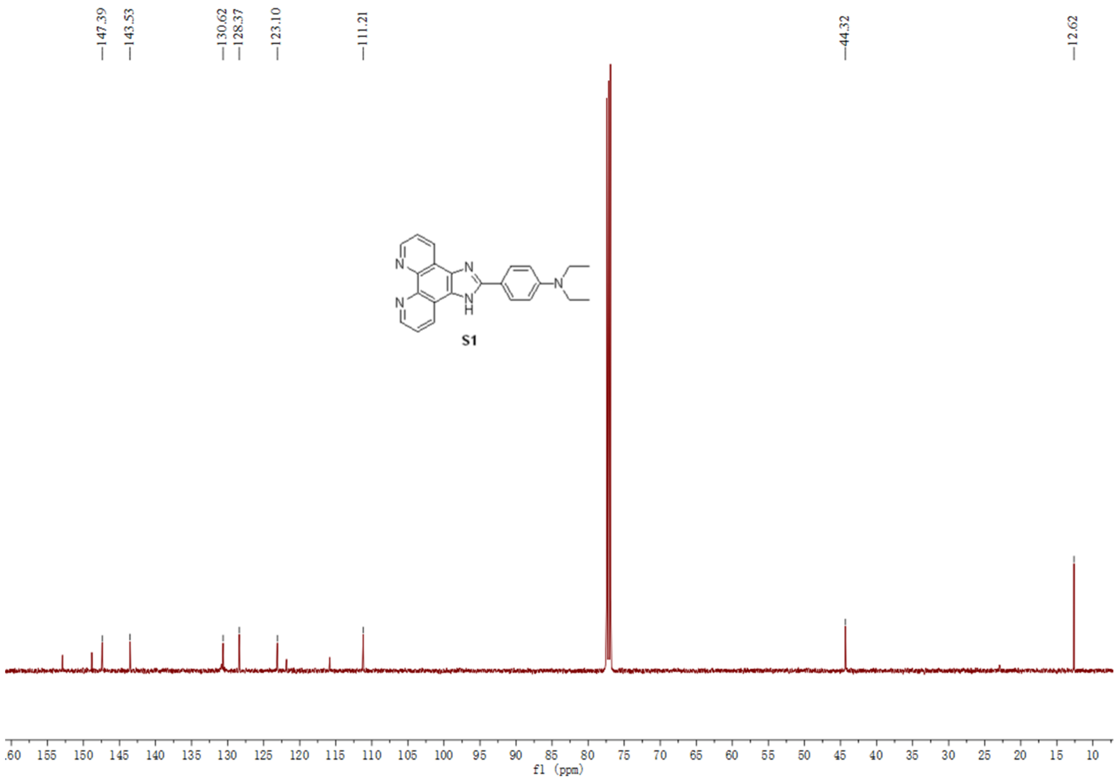

Figure S2. ^{13}C NMR spectrum of compound **S1**.

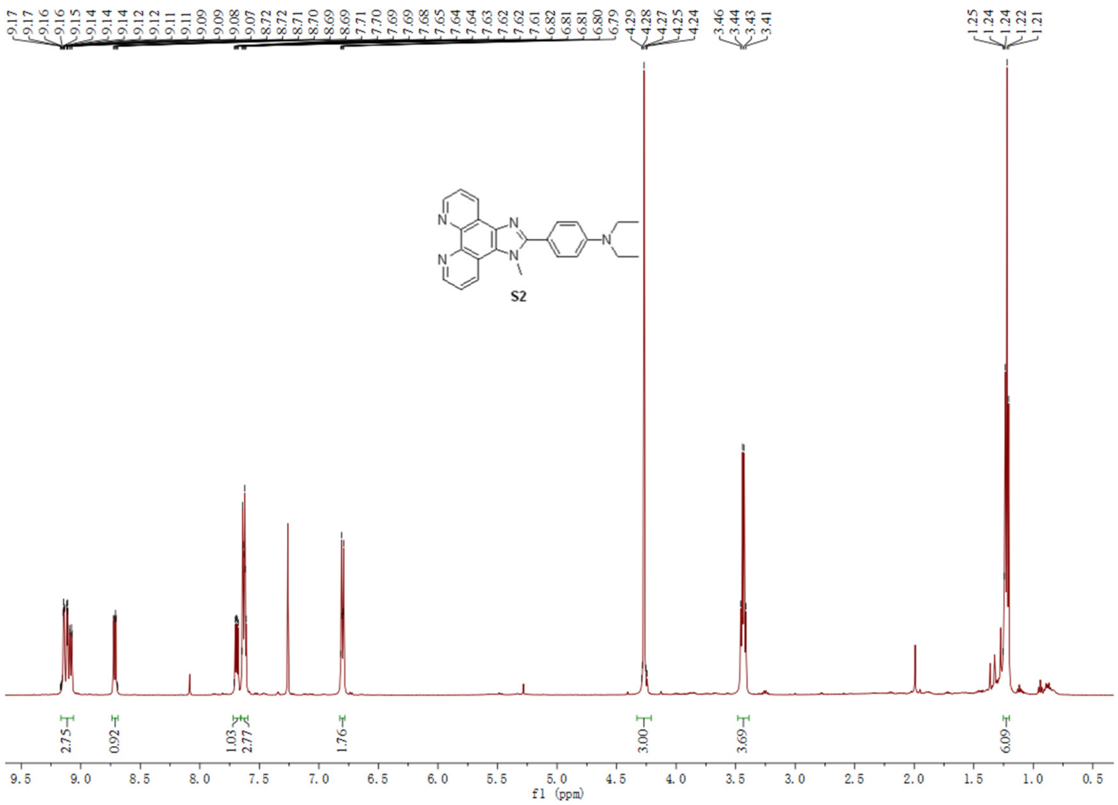

Figure S3. ^{1}H NMR spectrum of compound **S2**.

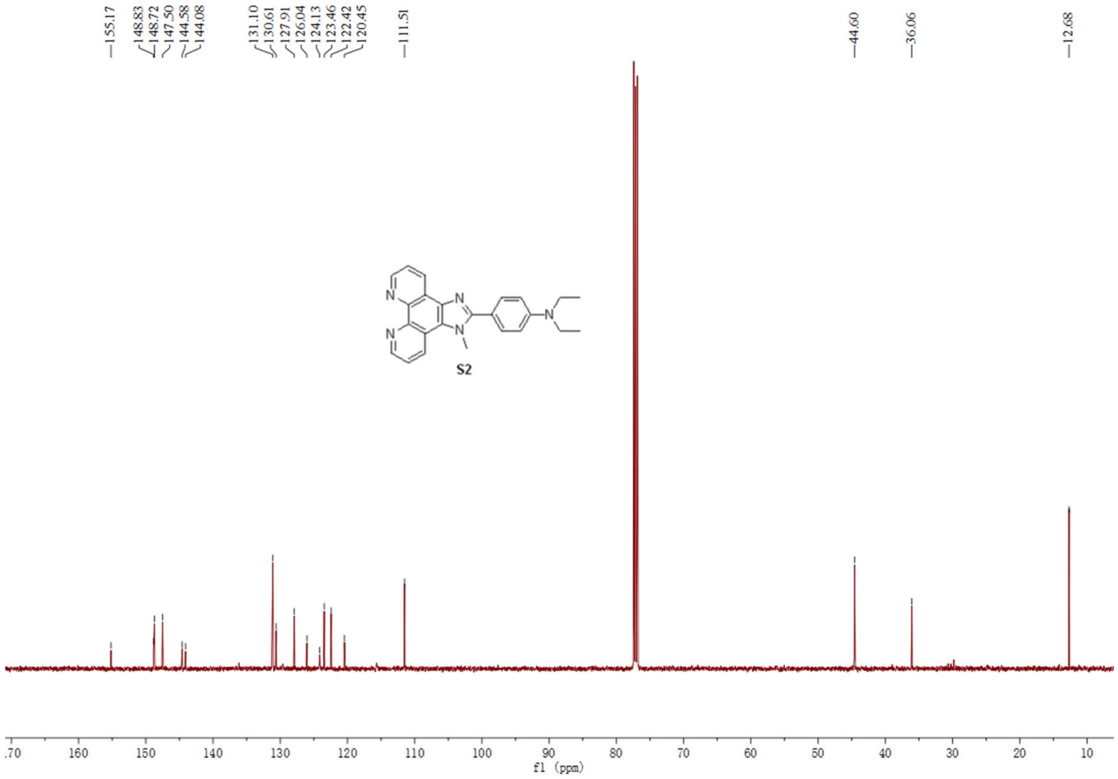

Figure S4. ^{13}C NMR spectrum of compound **S2**.

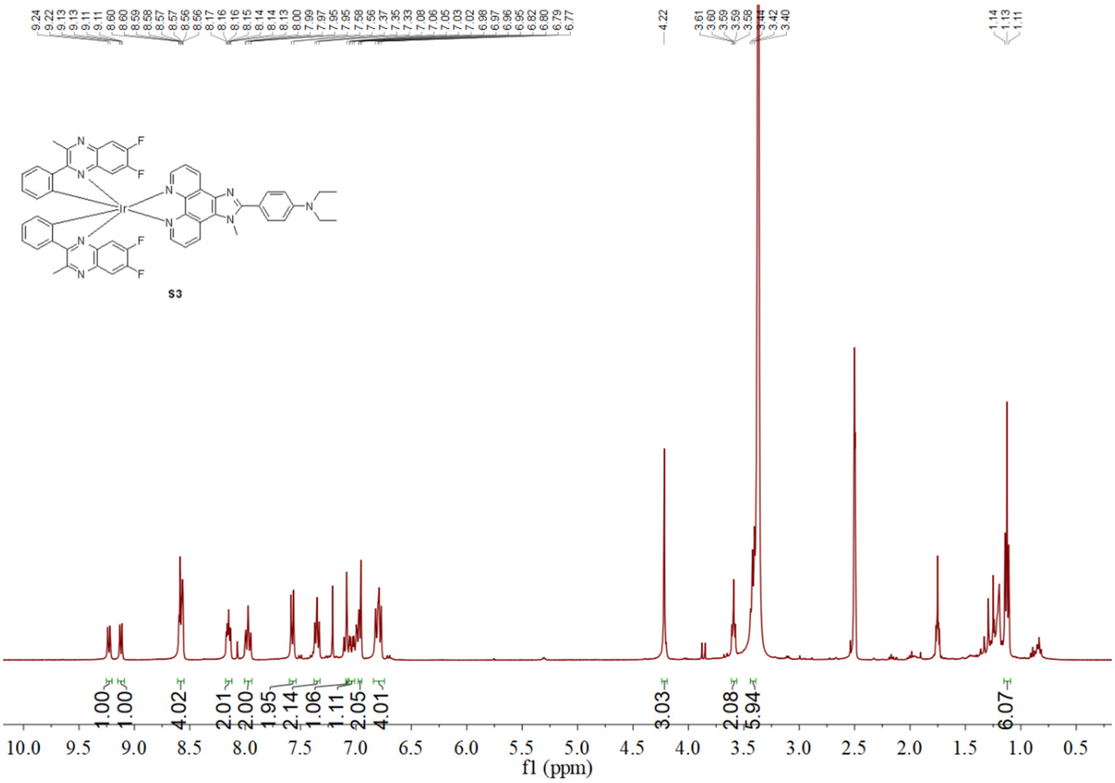

Figure S5. ^{1}H NMR spectrum of complex **S3**.

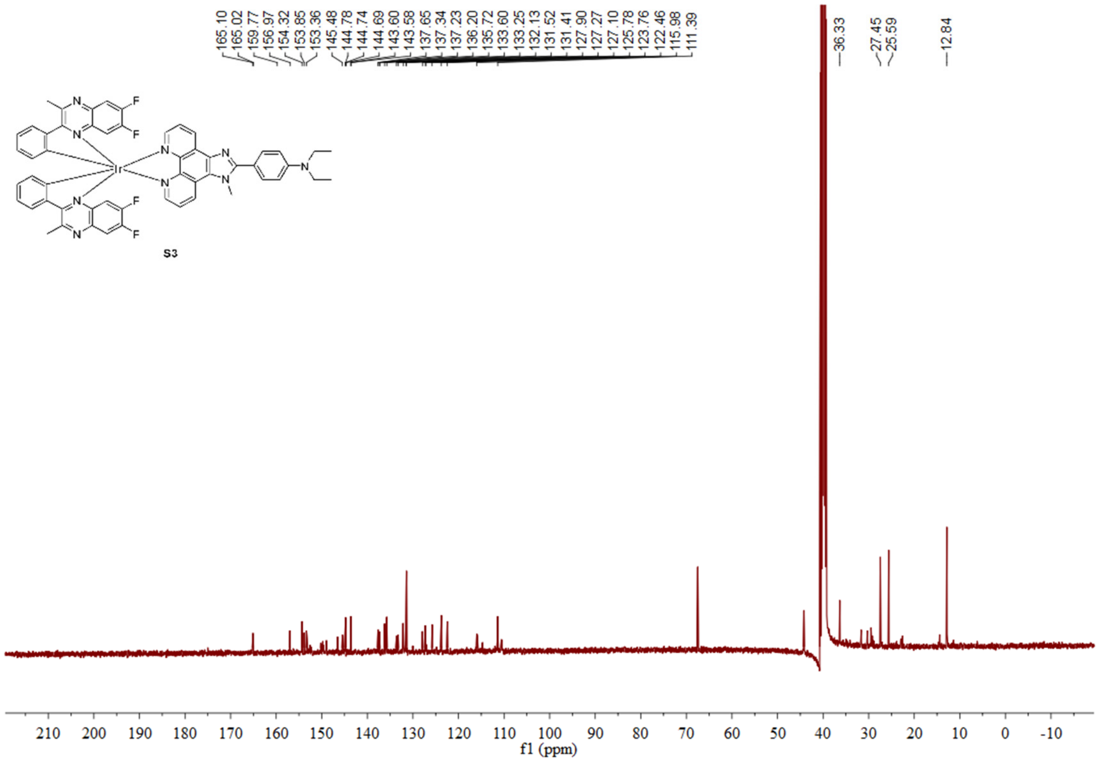

Figure S6. ^{13}C NMR spectrum of complex **S3**.

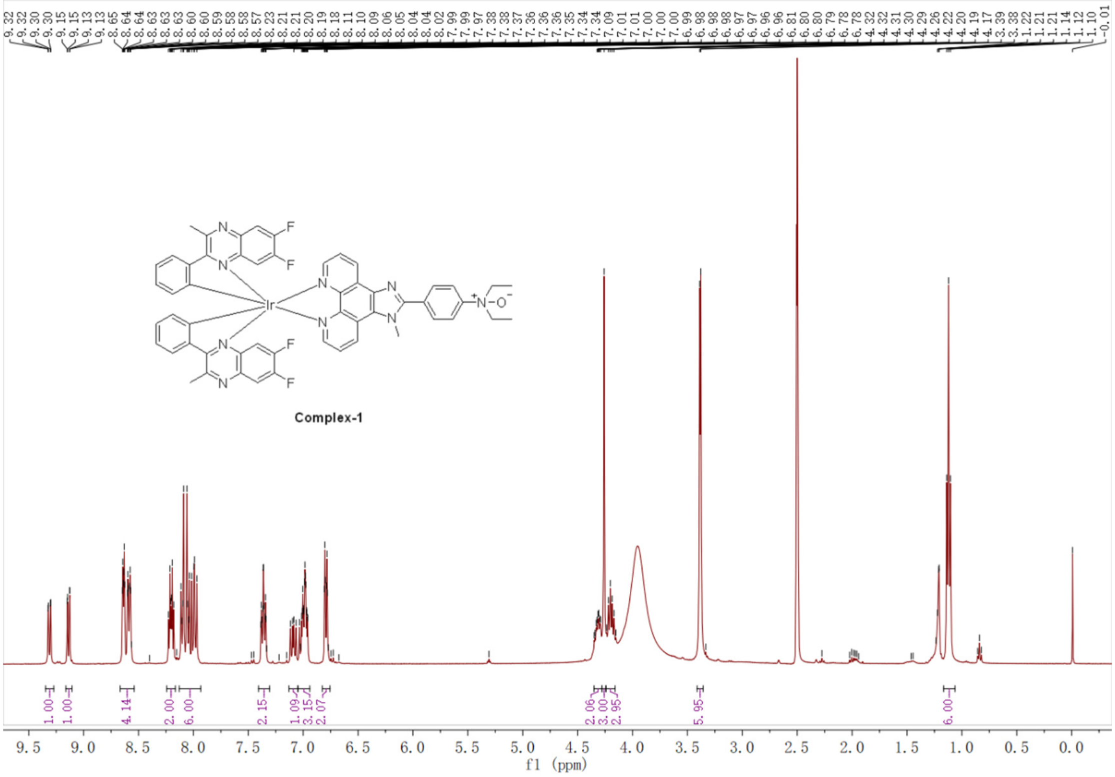

Figure S7. ^{1}H NMR spectrum of complex **1**.

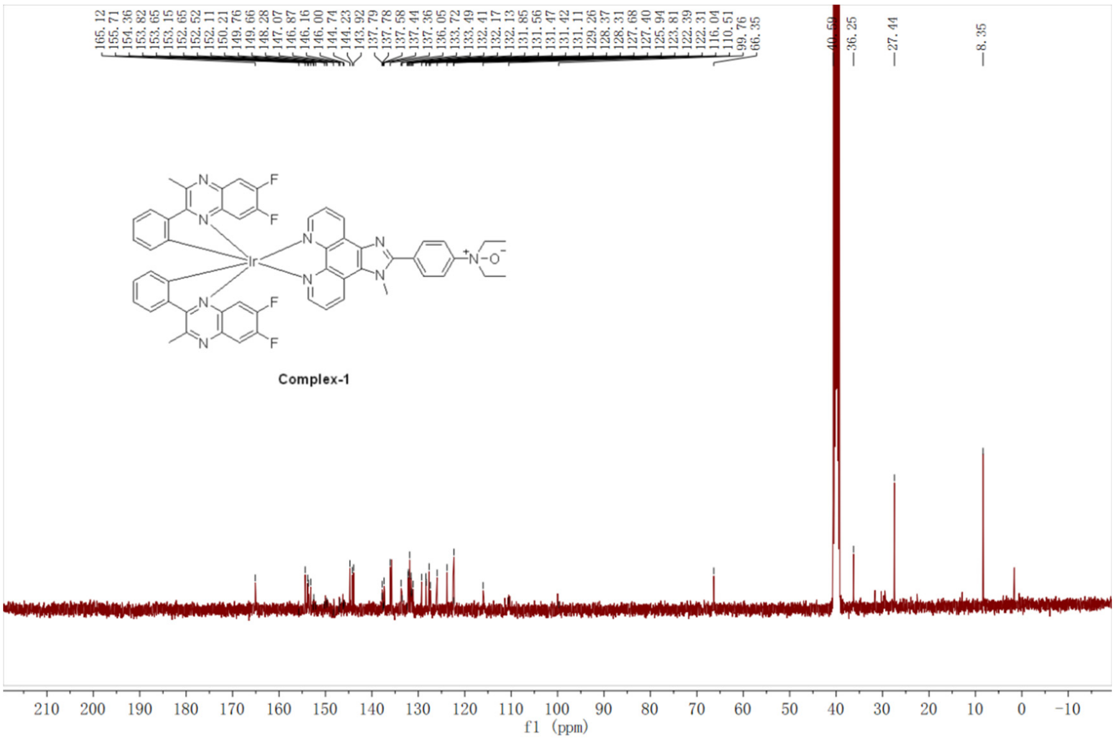

Figure S8. ^{13}C NMR spectrum of complex **1**.

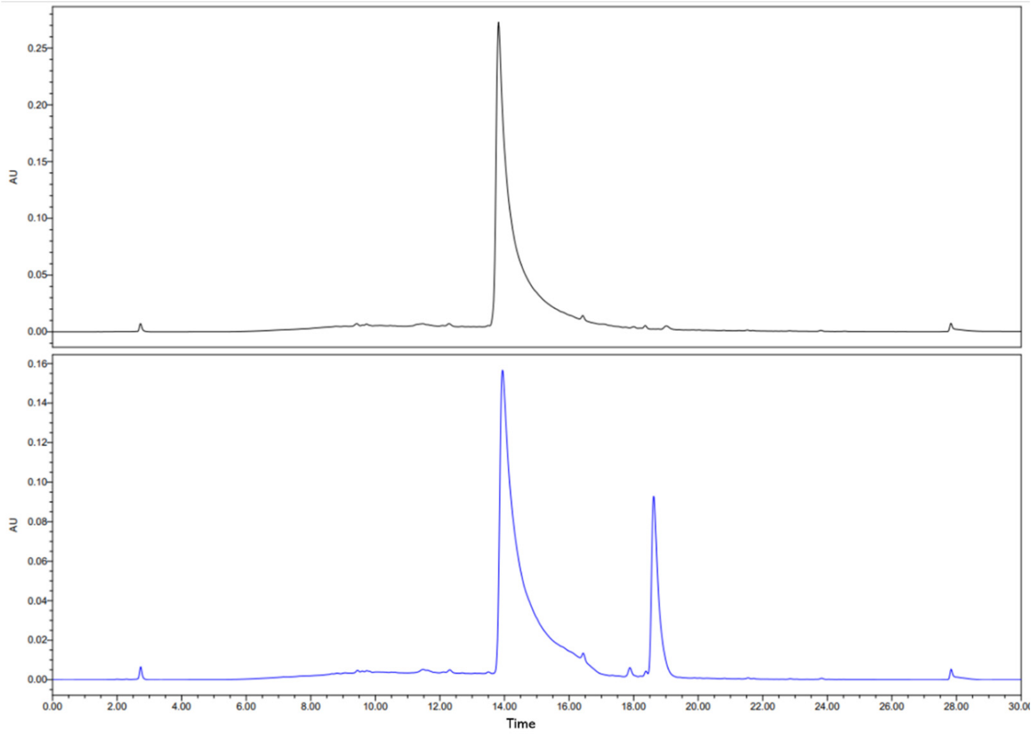

Figure S9. HPLC analysis of complex **1** (Upper) and complex **1** in the presence of Fe^{2+} ions (Lower). The absorbance was detected at 254 nm.

Part C: Detection experiment

Determination of LOD

Luminescence spectra of complex **1** to varying concentrations of Fe^{2+} ions (0−50 μM) in DMSO were recorded on a Hitachi U-3900 UV–vis Spectrophotometer under ambient temperature. To obtain the slope, the luminescence intensity at 675 nm was plotted against the concentration of Fe^{2+} ions (0.5−20 μM). Therefore, the detection limit was calculated according to the following formula based on 7 blank measurement:

$$LOD = 3\sigma/k$$

where σ is the standard deviation of the blank measurement and k is the slope between luminescence intensity and Fe^{2+} ions concentration

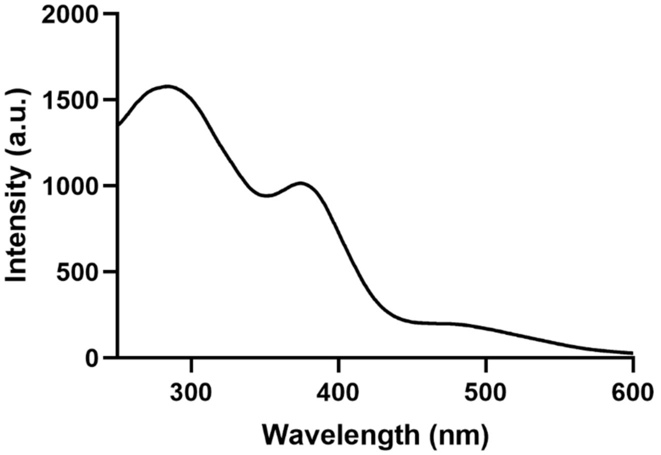

Figure S10. Excitation spectrum of complex **1** (10 μM) in DMSO. λ_{em} was set at 675 nm.

Table S1. Comparison of fluorescence probe/chemosensors for Fe^{2+} detection.

Detection method	Strategy	Target	Detection limit	Characteristics	Ref
**Fluorescence probe**	1,10-phenanthroline as turn-off fluorescence sensor	Fe^{2+}	2.74×10^{-7} M	Emission (512 nm), interfering with Cu(II) and Ni(II) ions, coordination binding mode	1
**Colorimetric probe**	Copper (I)-catalyzed 'Quick Click' generated linkers as sensors	Fe^{2+}	1×10^{-6} M 9.3×10^{-6} M	Low sensitivity, coordination binding mode	2
**Fluorescence probe**	Naphthalimide - based fluorescence sensor	Fe^{2+}	0.27×10^{-6} M	Emission (540 nm), reaction-based mode	3
**Dual-model probe**	Polyazomethine and ascorbic acid-based colorimetric and fluorescence probe	Fe^{2+}, Al^{3+}	1.85×10^{-7} M (fluorescence, Fe^{2+})	Emission (585 nm), coordination binding mode	4
**Fluorescence probe**	Coumarin-based fluorescence sensor	Fe^{2+}	0.8×10^{-7} M	Emission (488 nm), Reaction time (30 min) , reaction-based mode	5
**Luminescence probe**	NIR iridium(III) complexes-based luminescence probe	Fe^{2+}	5×10^{-7} M	Large Stokes shift (195 nm), rapid response (3 min) and NIR emission (675 nm)	This work

Table S2. Recoveries of complex **1** (20 μM) for the detection of Fe^{2+} ions in DMSO containing 2% Xi'an Qixiang Lake water.

Sample	Complex **1** (μM)	Fe^{2+} (μM)	Recovery (%)	RSD (%)
1	20	5	111.07	6.34
2	20	10	89.71	3.77
3	20	20	93.48	2.85

Reference

1. Arachchi, D. T.; Wijesekera, G.; De Costa, M.; Senthilnithy, R., Amino and chloro derivatives of 1, 10-phenanthroline as turn-off fluorescence sensors for selective and sensitive detection of Fe (II). *Journal of Photochemistry Photobiology A: Chemistry* **2020,** *402*, 112805.
2. Saini, P.; Singh, G.; Kaur, G.; Singh, J.; Singh, H., Copper (I)-catalyzed 'Quick Click'generated 1, 2, 3-triazole anthraquinone linkers for selective detection of Fe (II) ions. *Inorganic Chemistry Communications* **2022,** *141*, 109524.
3. Gupta, V. K.; Mergu, N.; Kumawat, L. K., A new multifunctional rhodamine-derived probe for colorimetric sensing of Cu(II) and Al(III) and fluorometric sensing of Fe(III) in aqueous media. *Sens. Actuators B Chem.* **2016,** *223*, 101-113.
4. Kamaci, U. D.; Kamaci, M.; Peksel, A., A dual responsive colorimetric sensor based on polyazomethine and ascorbic acid for the detection of Al (III) and Fe (II) ions. *Spectrochimica Acta Part A: Molecular Biomolecular Spectroscopy* **2021,** *254*, 119650.
5. Yang, C.; Xu, G.; Hou, C.; Peng, L.; Wang, W.; Zhang, H.; Zhang, X., A dual-mode nanoprobe based on silicon nanoparticles and Fe (II)-phenanthroline for the colorimetric and fluorescence determination of nitrite. *Microchim. Acta* **2023,** *190* (8), 318.
